# Associations of Prenatal Vitamin D status with Oral Health in Offspring: A Systematic Review

**DOI:** 10.3290/j.ohpd.b3505831

**Published:** 2022-10-20

**Authors:** Kornelija Rogalnikovaite, Egle Bendoraitiene, Vilija Andruskeviciene

**Affiliations:** a Student of Dentistry, Faculty of Dentistry, Lithuanian University of Health Sciences, Kaunas, Lithuania. Literature search, wrote the manuscript.; b Professor, Department of Preventive and Pediatric Dentistry, Lithuanian University of Health Sciences, Kaunas, Lithuania. Literature search, proofread the manuscript.; c Associate Professor, Department of Preventive and Pediatric Dentistry, Lithuanian University of Health Sciences, Kaunas, Lithuania. Proofread the manuscript.

**Keywords:** dental caries, developmental defects of enamel, pregnancy, vitamin D

## Abstract

**Purpose:**

The aim of this work is to evaluate the impact of prenatal vitamin D levels on oral health in offspring.

**Materials and Methods:**

The search was carried out in three databases: MEDLINE (PubMed), ResearchGate and Wiley Online Library. The inclusion criteria were randomised controlled trials and cohort studies published between June 16, 2017 and June 16, 2022, laboratory assessment of prenatal vitamin D status and evaluation of primary or mixed dentition for observation of dental caries and developmental defects of enamel. The risk of bias for randomised controlled trials was analysed according to the Cochrane risk-of-bias tool and Newcastle-Ottawa scale was used to assess risk of bias for cohort studies.

**Results:**

A total of 177 studies were identified, 11 were included in the data synthesis. Eight out of 11 studies were considered as high quality and the other 3 studies had moderate risk of bias. The synthesis of data revealed that the impact of prenatal vitamin D status on oral health in children is quite controversial and subsequent studies are necessary to examine whether vitamin D levels affect the risk of developing dental caries and enamel defects.

**Conclusion:**

The effect of prenatal vitamin D on oral health in offspring is not entirely clear. Since disturbances in dental hard tissues have a polyetiological origin, health specialists need to notify mothers about other possible risk factors and emphasise the importance of eating habits and individual oral hygiene in early childhood.

The formation of both primary and permanent teeth begins in utero and continues after birth.^16^ Development of tooth buds is determined by interactions of epithelial and mesenchymal cells and is strongly controlled by genetic, epigenetic, and environmental factors. In general, all the cells, tissues, organs, and organ systems function together to maintain homeostatic balance and a healthy state of the organism. Nutritional homeostasis certainly plays a relevant role in optimising health and preventing diseases. Nutritional health requires an adequate supply of essential nutrients such as carbohydrates, proteins, fats, water, vitamins, minerals, fiber, and other micronutrients to support life and longevity.^19^ Adequate nutrition is critical for oral health. Lack of minerals and vitamins is one of the risk factors that can lead to disturbance of the enamel developmental process.^8^

Vitamin D is involved in the homeostasis of calcium and phosphorus ions, which are essential for the mineralisation of tooth buds. Vitamin D deficiency or insufficiency in blood serum can induce hypocalcemia and hypophosphatemia. Deficient levels of calcium or phosphorus disrupt genetically determined dental calcification and maturation.^5^ Ameloblasts and odontoblasts are target cells of the active form of vitamin D (1,25–dihydroxyvitamin D), therefore the absence of vitamin D can cause developmental defects of enamel, such as as hypoplasia or hypomineralisation.^28^ Kühnisch et al^12^ confirmed that higher levels of vitamin D in blood serum negatively correlate with molar-incisor hypomineralisation (MIH). When the process of dental mineralisation is disturbed, the structure of enamel remains porous. Consequently, defects of dental hard tissues may predispose to dental caries. Dental caries is a lifestyle disease that results from oral microbiome dysbiosis. Factors that can facilitate an ecological shift of the microbiota are diet, regular toothbrushing,^27^ hyposalivation,^20^ host immune factors^38^ etc. However, the direct relation between prenatal vitamin D levels and dental caries is still inconclusive. Schroth et al^25^ were the first to reveal a significantly relevant association between lower prenatal vitamin D levels and higher decayed teeth score in infants. Tanaka et al^34^ noticed that high-dose vitamin D supplementation during pregnancy is related to reduced dental caries risk in children. Nevertheless, there is not enough data to prove the relationship between vitamin D intake and dental caries; thus, further epidemiological studies are needed to clarify the benefits of vitamin D supplementation.^29^

Nowadays vitamin D deficiency is a very common issue in Western societies. In recent years, vitamin D laboratory measurement and vitamin D intake have increased considerably.^2^ Several studies reported that majority of pregnant women have vitamin D deficiency and a strong correlation is observed when comparing prenatal and postnatal vitamin D levels.^11,39^ Insufficient postnatal vitamin D levels can disturb either primary or permanent teeth matrix secretion.

According to the National Academy of Medicine (formerly the Institute of Medicine), 50 nmol/l vitamin D level is considered to be a cut-off point for adequacy, but there is growing agreement between scientists that the concentration ≥ 75 nmol/l can provide protection against some negative health conditions, including early childhood caries and developmental defects of enamel.^26^ A few organisations suggest national or international guidelines on vitamin D supplementation during pregnancy. Recommended daily doses mostly vary from 400 to 600 IU of cholecalciferol, but these intakes are not approved by the World Health Organization (WHO).^7^ In 2020, WHO updated its own antenatal care guideline. In case of vitamin D deficiency during pregnancy, it is recommended to take 200 IU of vitamin D supplements per day.^40^ Even though vitamin D supplements are widely consumed in many European countries, various studies show that recommended doses do not ensure adequate blood serum vitamin D levels.^6^ For that reason, the aim of this work was to evaluate the impact of prenatal vitamin D status on oral health in offspring.

## Materials and Methods

### Protocol and Registration

The systemic analysis review report adhered to the Preferred Reporting Item for Systematic Review and Meta-Analyses (PRISMA) statement.^14^ The review was registered on the PROSPERO system under number CRD42022339729.

### Focus Question

The following focus question was developed according to the population, exposure, comparison, and outcome (PECO) study design (Table 1): Are deficient or insufficient prenatal vitamin D levels associated with a higher risk of early childhood caries, dental caries in mixed dentition and developmental defects of enamel compared with sufficient or optimal prenatal levels of vitamin D?

**Table 1:** PECO search strategy

Population	Pregnant women and their children with primary or mixed dentition
Exposure	Insufficient prenatal vitamin D levels (<50 nmol/l according to the National Academy of Medicine or <75 nmol/l according to the guidelines of Health Sciences Centre Winnipeg), assessed by laboratory analysis
Control	Sufficient or optimal prenatal vitamin D levels (>50 nmol/l or >75 nmol/l), assessed by laboratory analysis
Outcome	Associations between deficient or insufficient maternal vitamin D levels and early childhood caries, dental caries in mixed dentition and developmental defects of enamel

### Information Sources

The relevant articles were searched in the electronic databases MEDLINE (PubMed), ResearchGate and Wiley Online Library. Studies published between June 16, 2017, and June 16, 2022 were searched. The filter “dentistry” in the Wiley Online Library database was applied to minimise the number of articles that were not related to the subject. The manual search of additional relevant studies was performed revising of the bibliographies of full text articles.

### Search

The following keywords were used: “vitamin D”, “hypoplasia”, “hypomineralisation”, “dental caries”, “enamel defects”, “prenatal”, “maternal”, “pregnancy”, “childhood”. The search strategy is presented in Table 2.

**Table 2:** Search strategy

Search date	Database	Keywords	Articles
2022–06–16	PubMed Medline	(“Vitamin D”[All Fields] OR “Vitamin D”[MeSH Terms]) AND (“hypoplasia”[All Fields] OR “hypomineralisation”[All Fields] OR “dental caries”[MeSH Terms] OR (“dental”[All Fields] AND “caries”[All Fields]) OR “enamel defects”[All Fields]) AND (“prenatal”[All Fields] OR “maternal”[All Fields] OR “pregnancy”[All Fields] OR “childhood”[All Fields])	45
2022–06–16	ResearchGate	(“Vitamin D”) AND (“hypoplasia” OR “hypomineralisation” OR “dental caries” OR (“dental” AND “caries”) OR “enamel defects”) AND (“prenatal” OR “maternal” OR “pregnancy” OR “childhood”)	55
2022–06–16	Wiley Online Library	(“Vitamin D”) AND (“hypoplasia” OR “hypomineralisation” OR “dental caries” OR (“dental” AND “caries”) OR “enamel defects”) AND (“prenatal” OR “maternal” OR “pregnancy” OR “childhood”)	76

### Selection of Studies

Titles and abstracts of all identified studies and the full text of potentially eligible investigations were screened independently by two reviewers (K.R. and E.B.) based on the inclusion and exclusion criteria. Any disagreement over the eligibility of articles was resolved through discussion.

### Inclusion and Exclusion Criteria

The following inclusion criteria were applied:

- Prospective and retrospective cohort studies and randomised controlled trials (RCTs).
- Prenatal vitamin D levels were laboratory assessed.
- Dental examination provided by trained healthcare professionals.
- At least 1 year of follow-up.
- English language.
- Access to full text article.

The following exclusion criteria were applied:

- Systemic reviews, letters, dissertations, case reports, theses.
- Studies including children with systemic diseases.
- Studies evaluating the associations between umbilical cord blood and/or childhood vitamin D status and dental caries and/or developmental defects of enamel without analysing prenatal vitamin D levels.
- Subjective information on prenatal vitamin D status was collected through questionnaires and interviews.

### Data Extraction

From studies fulfilling the inclusion criteria, the following information was retrieved: author, year of publication, study design, study population, method for assessing prenatal vitamin D level, dental examination (follow-up), prevalence of dental caries, prevalence of developmental defects of enamel, and outcome.

### Risk of Bias Assessment

The methodological quality of studies was assessed during the data extraction process by two independent reviewers (K.R. and E.B). The risk of bias for randomised controlled trials was analysed according to the Cochrane risk-of-bias tool. The following items were evaluated as posing a low, high, or unclear risk of bias: 1) random sequence generation: 2) allocation concealment; 3) the blinding of participants and personnel; 4) blinding of outcome assessment; 5) incomplete outcome data; 6) selective reporting; 7) other bias. The degree of bias was categorised as low risk if all criteria were met, moderate risk when one criterion was missing, and high risk if two or more criteria were missing.

The Newcastle-Ottawa scale was used to assess risk of bias for cohort studies. The following domains were evaluated:

- Selection: a) representativeness of the exposed cohort; b) selection of the non-exposed cohort; c) ascertainment of exposure; d) demonstration that outcome of interest was not present at start of study based on the design or analysis.
- Comparability: cohorts are comparable on the basis of the design or analysis controlled for confounders.
- Outcome: a) assessment of outcome; b) adequate follow-up period for outcome of interest: c) adequacy of follow-up of cohorts.

The maximum score was 9 stars. Studies were classified into high risk (0–3 stars), moderate risk (4–6 stars) and low risk of bias (≥ 7 stars).

## Results

### Study Selection

**Fig 1:**
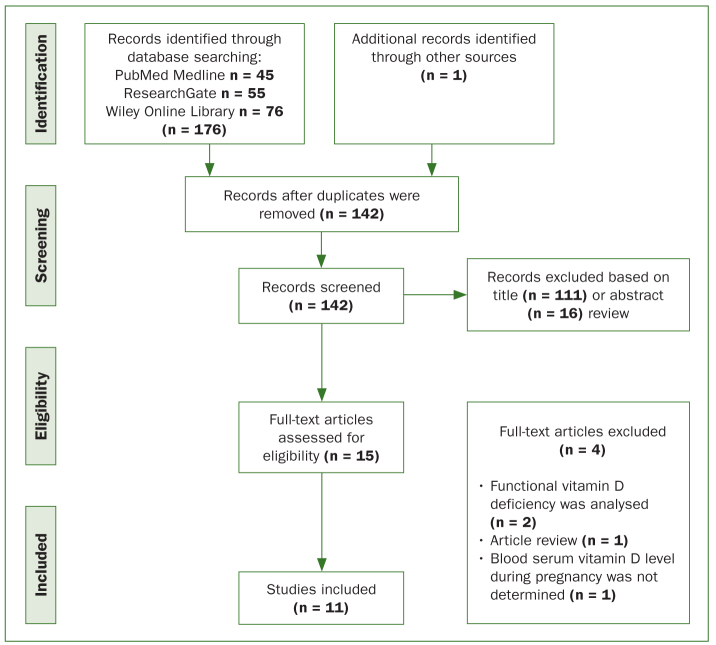
The Preferred Reporting Item for Systematic Review and Meta-Analyses (PRISMA) flow diagram.

From the search in the electronic databases, 176 records were identified and one more article was selected through a manual search. All records were exported to Zotero referencing software (Roy Rosenzweig Center for History and New Media, George Mason University; Arlington, VA, USA). After removing duplicates, 142 articles remained to be screened based on titles and abstracts. Fifteen studies were selected for full text analysis. After evaluation, four studies were excluded as they did not meet the inclusion criteria. The following reasons for excluding studies were: article review (n = 1);^36^ functional vitamin D deficiency was analysed (n = 2);^21,22^ prenatal vitamin D levels were not measured using laboratory methods (n = 1).^23^ Finally, 11 articles were included into the review. The study selection process is shown in Fig 1.

### Quality Assessment

Of the 11 studies included, 8 were considered as high quality (low risk of bias),^4,15,17,18,24,30,31,37^ while 3 cohort studies^3,32,33^ had a moderate risk of bias. The methodological quality analysis of the RCT and cohort studies is presented in Tables 3 and 4.

**Table 3:** Risk of bias assessment of the randomised controlled trial

Author, reference	Random sequence generation	Allocation concealment	Blinding of participants and personnel	Blinding of outcome assessment	Incomplete outcome data	Selective reporting	Other bias
Nørrisgaard et al, 2019^18^	+	+	+	+	?	+	?

+: low risk; ?: unclear risk; -: high risk.

**Table 4:** Risk of bias assessment of the cohort studies

Author, reference	Selection	Comparability	Outcome
Schroth et al, 2021^24^	***	**	**
Børsting et al, 2022^4^	***	*	***
van der Tas et al, 2019^37^	***	**	***
Navarro et al, 2021^17^	***	**	***
Silva et al, 2019^31^	***	**	**
Silva et al, 2019^30^	***	**	**
Suárez-Calleja et al, 2021^33^	***	*	**
Singleton et al, 2019^32^	***	*	*
Beckett et al, 2022^3^	***	*	**
Mortensen et al, 2022^15^	***	**	***

Selection. Representativeness of the exposed cohort: a) truly representative*; b) somewhat representative*; c) selected group, d) no description of the derivation of the cohort. Selection of the non-exposed cohort: a) drawn from the same community as the exposed cohort*; b) drawn from a different source: c) no description of the derivation of the non-exposed cohort. Ascertainment of exposure: a) secure record*; b) structured interview*: c) written self-report, d) no description, e) other. Demonstration that outcome of interest was not present at start of study: a) yes*; b) no.

Comparability. Comparability of cohorts on the basis of the design or analysis controlled for confounders: a) the study controls for socioeconomic status*; b) study controls for other factors: oral hygiene status, sugar and sweetened beverages consumption, maternal body mass index, low birthweight, alcohol consumption*; c) cohorts are not comparable on the basis of the design or analysis controlled for confounders.

Outcome. Assessment of outcome: a) independent blind assessment*; b) record linkage*; c) self-report, d) no description, e) other. Was follow-up long enough for outcomes to occur: a) yes (12 months)*; b) no. Adequacy of follow-up of cohorts: a) complete follow-up – all subjects accounted for*: b) subjects lost to follow up unlikely to introduce bias – number lost ≤20% or description of those lost suggested no different from those followed*; c) follow-up rate less than 80% and no description of those lost, d) no statement.

### Characteristics of Included Studies

The characteristics of included studies are described in Table 5. Of the 11 studies, one was an RCT^18^ and ten were cohort studies.^3,4,15,17,24,30-33,37^ Of these, one was retrospective^32^ and nine were prospective^3,4,15,17,24,30,31,33,37^ cohort studies. The majority of studies were carried out in Europe – the Netherlands,^17,37^ Norway,^4^ Denmark,^15,18^ Spain^33^ – with the others performed in the USA,^32^ Canada,^24^ New Zealand^3^ and Australia.^30,31^ The sample of population varied from 76^32^ to 5257^17^ mother-child pairs. In 7 studies,^3,4,15,17,24,32,37^ the vitamin D sufficiency cut-off point was considered to be 50 nmol/l, and in 1 study^33^ 75 nmol/l. In the study by Nørrisgaard et al,^18^ a mean serum vitamin D level assessed at the 24th week of pregnancy was approximately 75 nmol/l; we assume that it is a threshold value of sufficient prenatal vitamin D level. Silva et al^30,31^ analysed possible risk factors of caries and hypomineralised second primary molars (HSPM). To investigate associations between prenatal vitamin D levels and defects of dental hard tissues, logistic regression models with deficient vitamin D level equal to 20 nmol/l were fitted. Four studies^17,31-33^ investigated the association between prenatal vitamin D status and caries, five investigations^4,15,24,30,37^ studied the relationship between gestational vitamin D levels and developmental defects of enamel, and the remaining two studies^3,18^ analysed the association between prenatal vitamin D status and both caries and developmental defects of enamel.

**Table 5:** Description of included studies

Author, reference	Study design	Study population	Method for assessing prenatal vitamin D level	Dental examination (follow-up)	Prevalence of dental caries (or dmft/DMFT index scores)	Prevalence of developmental defects of enamel		Outcome
						Enamel hypoplasia	Enamel hypomineralisation	
Nørrisgaard et al, 2019^18^	Randomised controlled trial	496 mother-child pairs	LC-MS (24th pregnancy week)	After 6 years	2800 IU group: primary dentition: 20.1%; permanent dentition: 4.7%; permanent and/or primary dentition: 22.1%. 400 IU group: primary dentition: 20.2%; permanent dentition: 3.8%; permanent and/or primary dentition: 21%.	–	2800 IU group: primary dentition: 8.6%; permanent dentition: 15.1%; permanent and/or primary dentition: 18%. 400 IU group: primary dentition: 15.9%; permanent dentition: 27.5%; permanent and/or primary dentition: 29.4%.	No statistically significant association was found between high-dose vitamin D supplementation and dental caries in both primary (OR = 1.01, 95% CI = 0.65–1.59) and permanent (OR = 1.32, 95% CI = 0.41–4.48) dentition. Children whose mothers received high-dosage vitamin D supplements during pregnancy were statistically significantly more likely to have less DDE in both primary (OR = 0.54, 95% CI = 0.30–0.94) and permanent (OR = 0.42, 95% CI = 0.23–0.73) dentition.
Schroth et al, 2021^24^	Prospective cohort	Mothers: n = 134. Children: n = 135	Radioimmunoassay (second or early third trimester)	After 12 months	ECC in infants with EH: 73%. ECC in infants without EH: 27%.	DDE: 91%; EH: 22%	–	No statistically significant association was found between maternal vitamin D status and EH (p = 0.91). Infants with EH were statistically significantly more likely to be diagnosed with ECC compared with the children without EH (p < 0.001).
Børsting et al, 2022^4^	Prospective cohort	176 mother-child pairs	Electro-chemiluminescence immunoassay (18th–22nd and 32nd–36th pregnancy weeks)	After 7–9 years	–	–	Yellow/brown opacity on MIH or HSPM tooth: 17.6% (SD 0.81) PEB on MIH or HSPM tooth: 4.6% (SD 0.21) MIH: 31.8% (SD 1.74) HSPM: 22.2% (SD 0.98)	There was a statistically significant association between insufficient vitamin D levels (<50 nmol/l) assessed at weeks 18–22 and MIH (RR = 1.82, 95% CI = 1.13–2.93, p = 0.01).
van der Tas et al, 2018^37^	Prospective cohort	1840 mother-child pairs	LC-MS (18th–25th pregnancy weeks)	After 6 years	–	–	HSPM: 8.9% MIH: 8.1%	No statistically significant association was found between prenatal vitamin D status, MIH (OR = 1.05 per 10 nmol/l increase, 95% CI 0.98–1.12) and HSPM (OR = 1.02 per 10 nmol/l increase, 95% CI 0.98–1.07).
Navarro et al, 2021^17^	Prospective cohort	5257 mother-child pairs	LC-MS (18th–24th pregnancy weeks)	After 6 years	31.7%	–	–	Children whose mothers had prenatal vitamin D levels below 25 nmol/l and 50 nmol/l had statistically significant odds to be diagnosed with dental caries at the age of six (OR = 1.56, 95% CI = 1.18–2.06 and OR = 1.23, 95% CI = 1–1.5, respectively) compared with the children whose mothers had optimal vitamin D levels (≥75 nmol/l).
Silva et al, 2019^31^	Prospective cohort	172 mothers and 172 twin pairs	Chemiluminescent immunoassay (28th pregnancy week)	After 6 years	Any caries (noncavitated lesion and/or past treatment) – 32.2% Advanced caries (ICDAS 4-6 and/or past treatment) – 24.1%	–	–	No statistically significant association was found between maternal vitamin D status and dental caries in primary canines and molars (OR = 1.49, 95% CI = 0.9–2.45, p = 0.12).
Silva et al, 2019^30^	Prospective cohort	172 mothers and 172 twin pairs	Chemiluminescent immunoassay (28th pregnancy week)	After 6 years	–	–	HSPM: 19.6%	No statistically significant association was found between prenatal vitamin D levels and HSPM (OR = 1.17, 95% CI = 0.87–1.58, p = 0.3).
Suárez-Calleja et al, 2021^33^	Prospective cohort	Mothers: n = 178 Children: n = 188	High performance liquid chromatography (12th pregnancy week)	After 6–10 years	28.7%	–	–	Prenatal vitamin D levels below 50 nmol/l are a statistically significant risk factor of dental caries (OR = 2.51, 95% CI = 1.01–6.36, p = 0.029).
Singleton et al, 2019^32^	Retrospective cohort	76 mother-child pairs	Radioimmunoassay (6th–38th pregnancy weeks)	After 12–59 months	<50 nmol/l group: dmft 12–35 months: 9 (2.5); dmft 36–59 months: 14.4 (1.0) 50–75 nmol/l group: dmft 12–35 months: 7.4 (1.0); dmft 36–59 months: 10.1 (1.1)	–	–	There was no statistically significant difference in mean dmft scores for children aged 12 to 35 and 36 to 59 months whose mothers had insufficient and sufficient levels of vitamin D (p = 0.48 and p = 0.12, respectively).
Beckett et al, 2022^3^	Prospective cohort	81 mother-child pairs	LC-MS (third trimester)	After 5–6 years	55%	–	64%	Children whose mothers had insufficient vitamin D levels (<50 nmol/l) during the third pregnancy trimester had 3.6 times the rate of dental caries at the age of six compared with the children whose mothers had optimal vitamin D levels (≥ 50 nmol/l) (IRR = 3.55, 95% CI = 1.15–10.92, p < 0.05). No statistically significant association was found between insufficient maternal vitamin D levels, DDE prevalence (IRR = 0.37, 95% CI = 0.13–1.06) and severity (IRR = 0.47, 95% CI = 0.16–1.36).
Mortensen et al, 2022^15^	Prospective cohort	1241 mother-child pairs	LC-MS (<20th and ≥20th pregnancy week)	After 4 years	–	–	HSPM: 54.7% Yellowish/brownish demarcated opacities: 9.8% PEB: 2.8%	No statistically significant association was found between HSPM and maternal vitamin D levels measured in early and late pregnancy (p = 0.815 and p = 0.651, respectively).

DDE: developmental defects of enamel; ECC: early childhood caries; EH: enamel hypoplasisa; HSPM: hypomineralised second primary molar; ICDAS: International Caries Detection and assessment System; IRR: incidence rate ratio; LC-MS: liquid chromatography–mass spectroscopy; MIH: molar-incisor hypomineralisation; PEB: post-eruptive breakdown.

## Discussion

The present review of the relevant studies has shown conflicting evidence of prenatal vitamin D status on oral health in offspring. In three studies,^3,17,33^ a statistically significant association between prenatal vitamin D levels and caries was established. Suárez-Calleja et al^33^ verified that prenatal vitamin D concentration < 50 nmol/l is a risk factor for developing caries. Deficient vitamin D levels (<50 nmol/l) during pregnancy increase the risk of caries in permanent dentition 2.5 times. Similarly, Beckett et al^3^ observed that insufficient vitamin D concentrations (<50 nmol/l) increases the risk of caries in mixed dentition 3.6-fold at the age of six. Navarro et al^17^ found out that 6-year-old children whose mothers had severe prenatal vitamin D deficiency and deficient prenatal vitamin D concentrations were more likely to be diagnosed with caries compared with the children whose mothers had optimal (>75 nmol/l) vitamin D concentration. In the remaining three studies,^18,31,32^ no statistically significant evidence was found to support a link between prenatal vitamin D status and caries incidence in children. It is important to note that Suárez-Calleja et al^33^ and Beckett et al^3^ enrolled a small number of mother-child dyads – 76 and 81, respectively. Furthermore, the Suárez-Calleja et al^30^ study was considered to have moderate risk of bias. Potentially, these features may affect the results. Even though Navarro et al^17^ found a statistically significant association between prenatal vitamin D levels and caries risk, the authors reported that evidence is weak. Scoring caries on intraoral photographs has been validated as an alternative diagnostic method to visual and tactile examination,^9^ but researchers presume that this technique may fail to detect carious lesions.

Nørrisgaard et al^18^ and Børsting et al^4^ observed a statistically significant relationship between prenatal vitamin status and developmental defects of enamel. Nørrisgaard et al^18^ reported that high-dosage vitamin D supplementation during pregnancy was associated with 50% reduced odds of enamel defects at the age of six. In addition, hypomineralised lesions on primary second molars (HPSM) can be considered as a predictive sign of MIH, because children diagnosed with DDE (developmental defects of enamel) in the primary dentition were more likely to also have DDE in the permanent dentition. Currently, little is known about etiological variables and their interaction during the enamel developmental process, so that no preventive efforts for enamel defects are available. The authors suggest prenatal vitamin D supplementation as an essential healthcare strategy for the prevention of the disease. A daily dose of 2800 IU may seem too high, but several studies have verified that a daily vitamin D intake of up to 4000 IU is safe and should not lead to hypervitaminosis D and its resulting toxicity.^13,35^ Børsting et al^4^ found that an insufficient vitamin D concentration measured at 18–22 weeks is associated with MIH, but no statistically significant differences regarding HSPM were identified. In this cohort study, many participants were lost to follow-up: among 841 mothers who agreed to participate in the study, a total of 176 children were included in the data analysis. The authors pointed out that low follow-up rate is the main limitation of their study. The remaining four investigations^15,24,30,37^ did not demonstrate a statistically significant relationship between prenatal vitamin D status and DDE.

The analysis of the included studies showed that a variety of methods for assessing vitamin D levels was used. Based on the studies, liquid chromatography-mass spectrometry (LC-MC) is a reference standard to quantitatively determine vitamin D levels in blood serum. LC-MC provides an accurate measurement of total 25-hydroxyvitamin D (25(OH)D) that improves monitoring of vitamin D reserves in high-risk patients.^1^ In five^3,15,17,18,37^ out of eleven studies, prenatal vitamin D levels were analysed using liquid chromatography-mass spectrometry: we assume that these studies have less bias in the measurement of serum 25(OH)D concentrations.

As we mentioned before, in the cohort study by van der Tas et al,^37^ MIH and HSPM were scored from intraoral radiographs. Some research studies claim that a radiographic examination is suitable for detecting advanced dental hard tissue defects, while other possible diagnostic methods should be considered for detecting caries lesions at the initial stage. For example, visual and tactile examination was found to have high accuracy and specificity.^10^ Although researchers in van der Tas et al^37^ were trained to produce high-quality radiographs, some of the participating children were excluded from the study because low-quality radiographs made it impossible to evaluate MIH and HSPM prevalence. Moreover, as in the Navarro et al^17^ study, information bias may be inherent in the radiographic method, since lesions in dental hard tissues can be underestimated or misclassified. Nevertheless, van der Tas et al^37^ stated that these limitations presumably did not affect their results.

To evaluate hypomineralisation defects, the number of erupted permanent teeth is essential. Participants in the van der Tas et al study^37^ underwent digital intraoral radiographic examinations at a mean age of six. 62.5% of participating children had incomplete image sets or low-quality radiographs to score MIH and were not included in the data analysis. A smaller sample size for MIH led to a higher prevalence of HSPM (8.9% vs 8.1%). Consideration of adequate follow-up time is necessary to avoid some risk of bias. In the Børsting et al study,^4^ children were dentally examined at the age of 7–9. Most of the children were examined at the age of 8. According to those authors, the prevalence of MIH was quite high (31.8%) compared to previous investigations. The high prevalence of MIH in that study may be explained by methodological factors, as the dental examination was carried out based on recommendations regarding the age at which both first permanent molars and incisors must be fully erupted. In the study conducted by Nørrisgaard et al,^18^ 66.9% of children had at least 1 fully erupted first permanent molar and 47.2% of six-year-old participants had all four. There was no relationship between vitamin D intake in high doses and eruption of permanent molars; therefore, the inclusion of children who did not have complete set of permanent first molars may not have biased the results.

Caries and developmental defects of enamel are disturbances in dental hard tissues that have a multifactorial origin. Although the main objective of the included studies was prenatal vitamin D status, the authors investigated other conditions that increase the chance of the occurrence of caries and enamel defects. Schroth et al^24^ reported that low maternal calcium levels, lack of knowledge about vitamin D, infrequent milk and margarine intake were significantly associated with enamel hypoplasisa (EH). Silva et al^31^ found that HSPM, lack of community water fluoridation, and maternal obesity were early-life environmental risk factors for caries,^31^ and that socioeconomical status, maternal smoking later in pregnancy, and vitamin D deficiency at birth were crucial factors in HSPM etiology.^30^ Mortensen et al^15^ found that lower gestational age and higher maternal education (high school and ≥ 1 year of further education) were associated with a higher risk of HSPM. Interestingly, a statistically significant association between HSPM and higher education was observed only among primiparous mothers. Suárez-Calleja et al^33^ emphasised the influence of behavioural factors on the prevalence of caries. Those authors established a statistically significant relationship between the presence of caries, toothbrushing technique and frequency of sugar intake. Incorrect brushing technique and regular sugar consumption increased the risk of caries in Spanish children by approximately threefold.^33^ Singleton et al^32^ found no statistically significant relationship between breastfeeding and higher dmft index scores. Those authors noted that the behavioural risk factors (oral hygiene, intake of sugar-sweetened beverages), in addition to other contributors, exert a strong influence on caries when children get older. Therefore, they plan to conduct another study that will cover several potentially confounding factors.

To summarise, the current analysis of the included studies showed insufficient evidence that low prenatal vitamin D levels can lead to higher prevalence of caries and enamel defects in offspring. Silva et al^28^ studied the association between prenatal and childhood vitamin D levels and caries in children. Those authors pointed out that insufficient prenatal vitamin D concentration (<75 nmol/l) should be considered as one of the possible caries risk indicators. The authors of the studies included here mention some limitations of their studies. Consequently, long-term observational studies or clinical trials are needed to obtain reliable proof to validate causality between vitamin D supplementation during pregnancy and oral health in children.

### Limitations

Ten out of 11 investigations included in our review are cohort studies. Since the participants of cohort studies can be exposed to other risk factors that also influence the outcome of interest, confounding may occur. The diversity of laboratory methods for assessing prenatal vitamin D levels, the variety of methodologies to accomplish a dental examination, different cut-off points of vitamin D status, participant characteristics such as age, sample size, the phase of dentition, the number of erupted teeth, and follow-up rate caused heterogeneity among the included studies. For all these reasons, no meta-analysis was performed.

## Conclusion

The role of prenatal vitamin D levels on oral health in offspring is not entirely clear. To avoid possible adverse outcomes caused by vitamin D deficiency, interprofessional collaboration between general practitioners, midwives and oral health professionals is required. Since lesions in dental hard tissues have polyetiological origins, health specialists need to notify mothers about other possible risk factors and emphasise the importance of eating habits and individual oral hygiene in early childhood.
